# High efficacy of third generation EGFR inhibitor AZD9291 in a leptomeningeal carcinomatosis model with *EGFR*-mutant lung cancer cells

**DOI:** 10.18632/oncotarget.6758

**Published:** 2015-12-24

**Authors:** Shigeki Nanjo, Hiromichi Ebi, Sachiko Arai, Shinji Takeuchi, Tadaaki Yamada, Satsuki Mochizuki, Yasunori Okada, Mitsutoshi Nakada, Takashi Murakami, Seiji Yano

**Affiliations:** ^1^ Department of Medical Oncology, Kanazawa University Cancer Research Institute, Kanazawa, Japan; ^2^ Department of Pathology, Keio University School of Medicine, Tokyo, Japan; ^3^ Department of Neurosurgery, Graduate School of Medical Science, Kanazawa University, Kanazawa, Japan; ^4^ Laboratory of Tumor Biology, Takasaki University of Health and Welfare, Takasaki, Japan

**Keywords:** leptomeningeal carcinomatosis, EGFR-TKI resistance, EGFR mutation, EGFR inhibitors

## Abstract

Leptomeningeal carcinomatosis (LMC) remarkably decreases the quality of life of *EGFR*-mutant lung cancer patients. In contrast to the lesions outside the central nervous system (CNS), molecular mechanisms of EGFR tyrosine kinase inhibitor (TKI) resistance in CNS lesions including LMC are largely unknown. In this study, we established an *in vivo* imaging model for LMC with *EGFR* mutant lung cancer cell lines harboring an exon 19 deletion in *EGFR* and evaluated the effect of first generation EGFR-TKIs, erlotinib, second generation afatinib, and third generation AZD9291. In PC-9/ffluc model, erlotinib treatment slowed the development of LMC. Importantly, treatment with afatinib or AZD9291 apparently delayed the development of LMC. Moreover, treatment with a higher dose of AZD9291, also associated with inhibited phosphorylation of EGFR downstream molecule S6, regressed LMC refractory to the aforementioned EGFR-TKI treatments. These observations suggest that the third generation EGFR-TKI AZD9291 may be an effective treatment for first or second generation EGFR-TKI resistant LMC caused by *EGFR*-mutant lung cancer.

## INTRODUCTION

Central nervous system (CNS) metastasis including brain metastasis and leptomeningeal carcinomatosis (LMC) often develops in several types of cancers such as lung, breast, and renal cancer. It is a grave complication that shortens survival and markedly diminishes the quality of life of the patients [1]. The epidermal growth factor receptor (EGFR)-mutant-lung cancer represents 10% and 25% of non-small-cell lung cancers (NSCLC) in Caucasians and East Asians, respectively [2]. LMC develops in patients that have the *EGFR* mutation more often compared with patients without the mutation [3]. EGFR-tyrosine kinase inhibitors (EGFR-TKIs) such as first generation gefitinib/erlotinib and second generation afatinib showed remarkable activity in *EGFR*-mutant lung cancer patients with or without CNS metastasis [4, 5, 6]. However, EGFR-TKI resistance may eventually develop after varying periods of treatment, and approximately one-third of patients develop CNS metastases after acquisition of EGFR-TKI resistance [7–9]. Brain metastases are manageable by concomitant use of EGFR-TKI and radiation therapy including whole brain irradiation and stereotactic radiotherapy [10]. There is, however, no established therapy for LMC, which is resistant to first and second generation EGFR-TKIs. Therefore, novel and effective therapies need to be developed for managing LMC in cancer patients.

Third generation EGFR-TKIs such as AZD9291 and CO-1686 effectively block EGFRs with the T790M mutation as well as exon 19 deletion and L858R-sensitive mutations, but do not inhibit wild type EGFRs. They have also shown clinical efficacy in *EGFR*-mutant lung cancer patients who previously treated with EGFR-TKIs [11, 12]. However, the efficacy of third generation EGFR-TKIs on CNS metastasis is not well reported in literature.

In the present study, we established an *in vivo* imaging model for LMC with an EGFR-TKI sensitive *EGFR*-mutant lung cancer cell lines. Furthermore, we evaluated the efficacy of AZD9291 in comparison with erlotinib and afatinib in our LMC model.

## RESULTS

### Establishment of an *in vivo* imaging model for LMC of *EGFR*-mutant lung cancer

**Figure 1:**
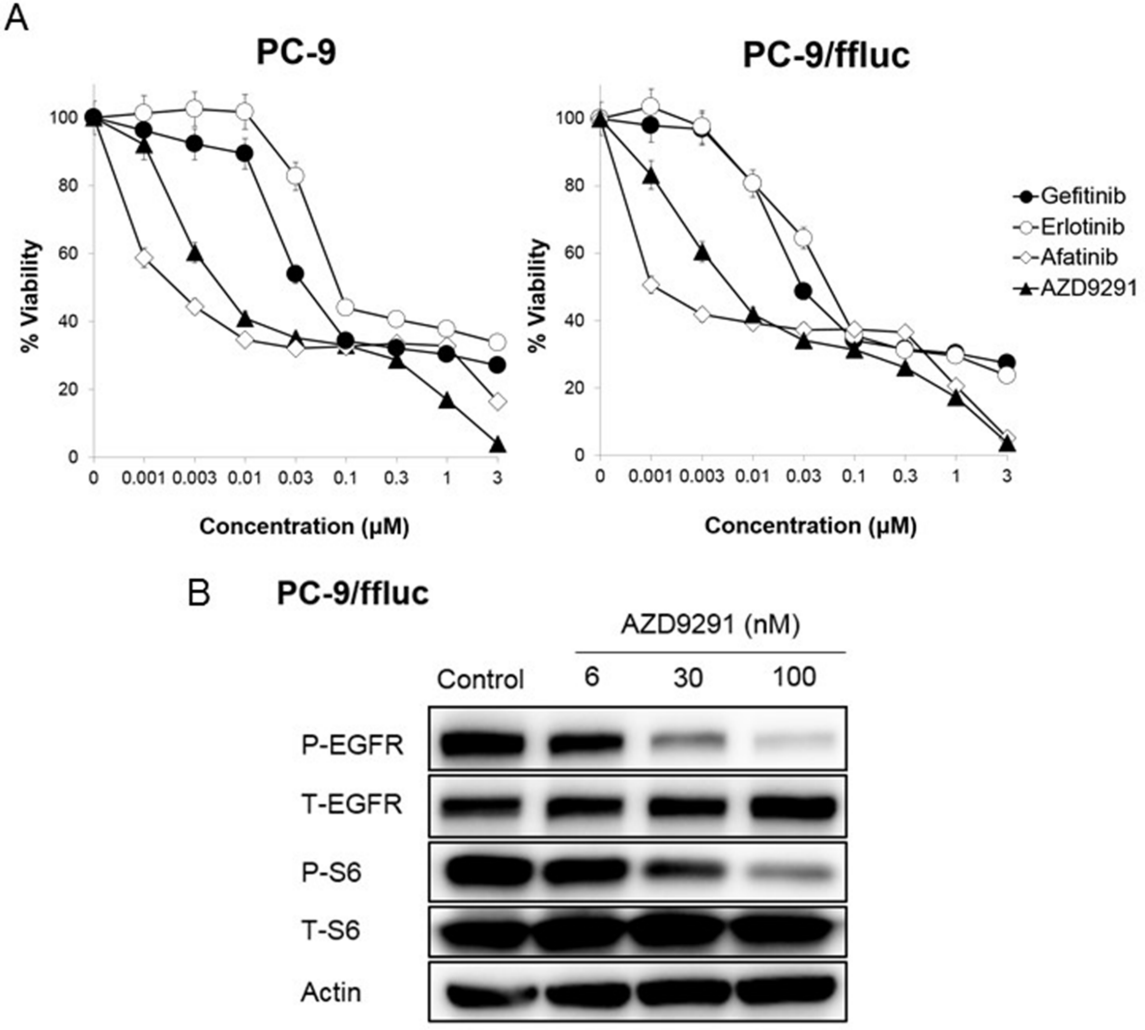
Sensitivity of PC-9/ffluc cells to EGFR-TKIs *in vitro* (**A**) PC-9, and PC-9/ffluc cells (2 × 10^3^ cells per well) were incubated with various concentrations of erlotinib, gefitinib, AZD9291, and afatinib for 72 hours. Cell growth was determined by the MTT assay. Bars represent SD. (**B**) PC-9/ffluc cells were incubated with AZD9291 (6, 30, 100 nmol/L) for 24 hours. The cell lysates were harvested and phosphorylation of indicated proteins was determined by western blot analysis. Data shown are representative of three independent experiments with similar results.

**Figure 2:**
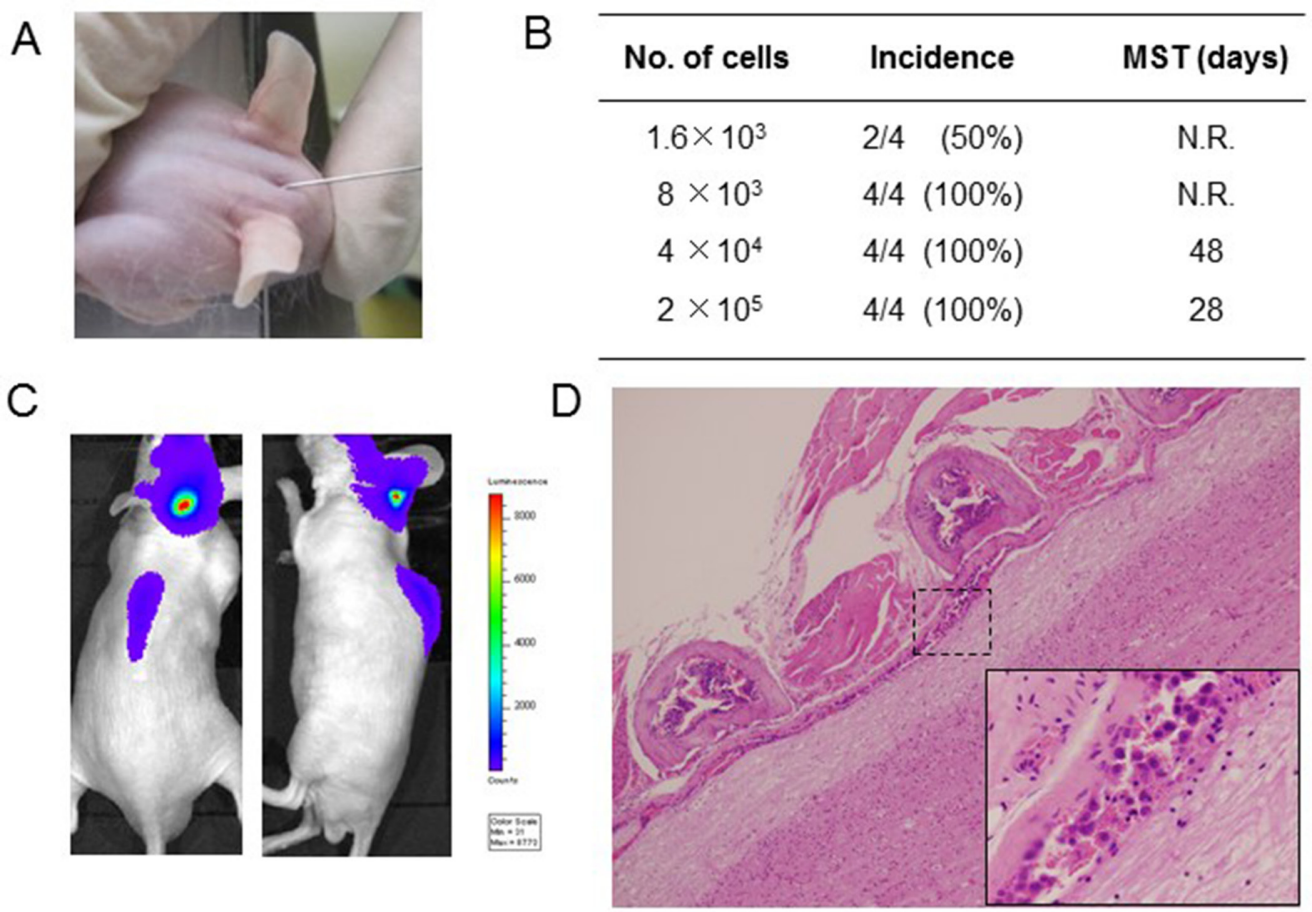
Leptomeningeal carcinomatosis model with PC-9/ffluc cells (**A**) PC-9/ffluc cells were inoculated into the space between the external occipital protuberance and first cervical vertebra of SHO-SCID mice. (**B**) Production of LMC was determined using the IVIS imaging system; MST: median survival time; N.R: not reached. (**C**) Representative imaging of mice immediately after PC-9/ffluc cell inoculation. (**D**) Representative histological images of spinal cord with LMC (x40). The high-power field image of dotted area is shown in the lower right corner (x400).

To establish an LMC model with an *EGFR*-mutant lung cancer, we utilized the human *EGFR* mutant lung adenocarcinoma PC-9/ffluc (exon 19 deletion) [13], HCC827/luc, and H1975/luc cells, which were transfected with the fusion gene of luciferase for *in vivo* imaging. EGFR-TKIs such as gefitinib, erlotinib, afatinib, and AZD9291 decreased viability of PC-9/ffluc and HCC827/luc cells (Figure 1A, Supplementary Figure 4). On the other hand, only afatinib and AZD9291 decreased viability of H1975/luc cells (Supplementary Figure 1). There was no discernible difference between luciferase-gene transfectants and parental cells, in terms of sensitivity to EGFR-TKIs. Western blot analysis of PC-9/ffluc revealed that AZD9291 inhibited phosphorylation of EGFR and its downstream molecule S6 in a dose-dependent manner (Figure 1B). We inoculated PC-9/ffluc cells into the leptomeningeal space of SHO-SCID mice (Figure 2A). PC-9/ffluc cells (more than 1.6 × 10^3^) developed LMC in SHO-SCID mice. The survival of the recipient mice was shortened in a cell number-dependent manner (Figure 2B). All mice inoculated with 2 × 10^5^ PC-9/ffluc cells developed LMC (Figure 2C, 2D) and became moribund within 28 days; we used this experimental protocol for the following experiments.

### Effect of EGFR-TKIs in the LMC model

**Figure 3:**
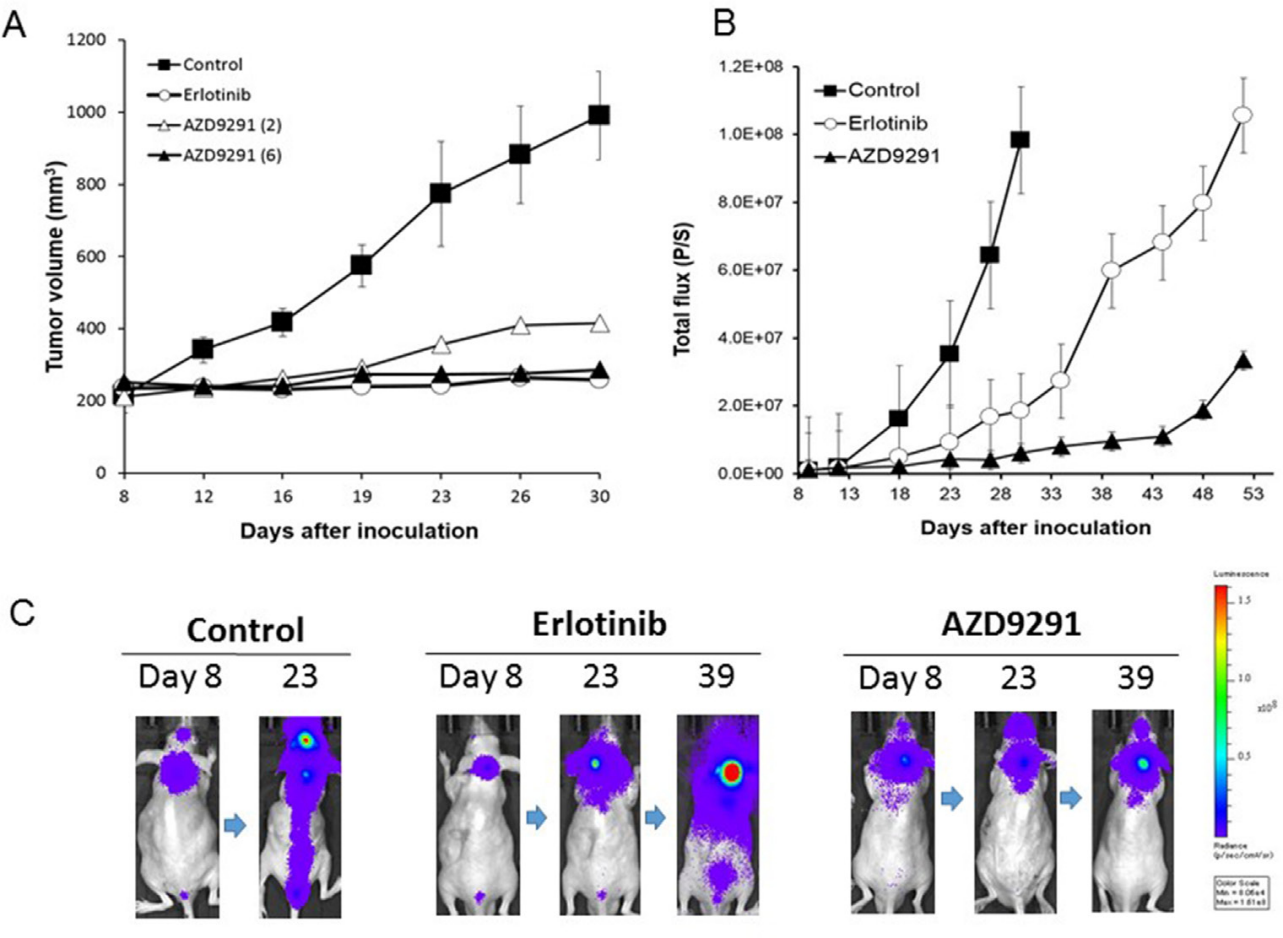
Effect of EGFR-TKI treatment in subcutaneous and LMC models with PC-9/ffluc cells (**A**) PC-9/ffluc cells were inoculated subcutaneously into SHO-SCID mice (*N* = 4). Daily oral treatment with erlotinib (25 mg/kg) or AZD9291 (6 mg/kg) was given from day 8 until day 30. (**B**) PC-9/ffluc cells were inoculated into the leptomeningeal space of SHO-SCID mice (*N* = 5). Daily oral treatment with erlotinib (25 mg/kg) or AZD9291 (6 mg/kg) was given from day 5 until day 50. Bars represent SD. (**C**) Representative images of mice treated with or without erlotinib (25 mg/kg) or AZD9291 (6 mg/kg).

We sought to examine the effect of AZD9291 in our LMC model, as compared with erlotinib. In preliminary experiments with the subcutaneous tumor model, daily oral treatment with 25 mg/kg of erlotinib remarkably prevented the enlargement of PC-9/ffluc tumors for more than 23 days. In the same subcutaneous model, daily oral treatment with 6 mg/kg of AZD9291 also prevented the enlargement of PC-9/ffluc tumors (Figure 3A). Therefore, we determined that 25 mg/kg of erlotinib and 6 mg/kg of AZD9291 have equivalent activity in our mouse model.

**Figure 4:**
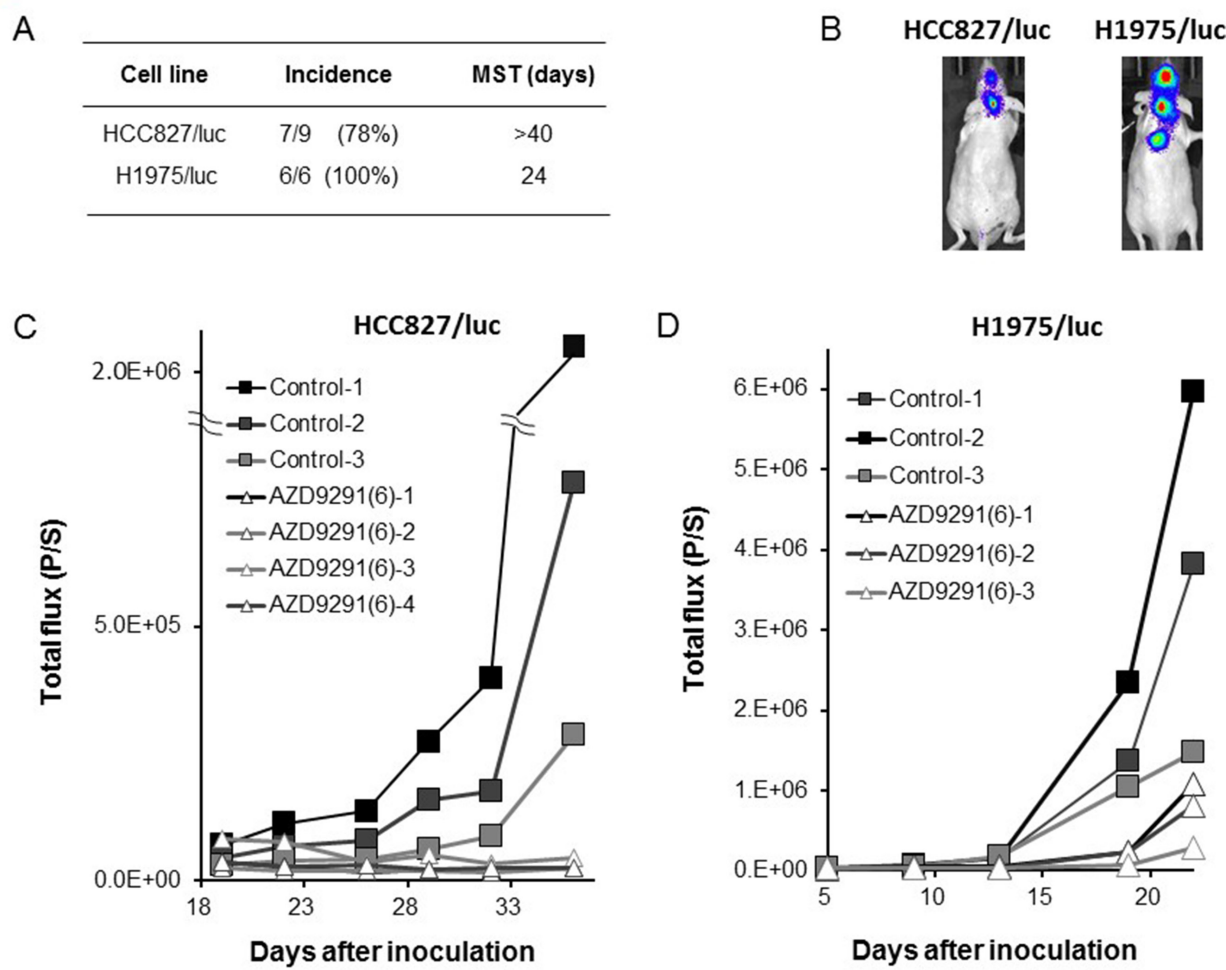
Effect of EGFR-TKI treatment in LMC models with HCC827/luc and H1975/luc cells HCC827/luc and H1975/luc cells were inoculated into the space between the external occipital protuberance and first cervical vertebra of SHO-SCID mice. (**A, B**) Production of LMC was determined using the IVIS imaging system; MST: median survival time. (**C**) The mice inoculated with HCC827/luc cells were daily treated with control (*N* = 3) or AZD9291 (6 mg/kg) (*N* = 4) from day 18 until day 36. (**D**) The mice inoculated with H1975/luc cells were daily treated with control (*N* = 3) or AZD9291 (6 mg/kg) (*N* = 3) from Daily oral treatment with AZD9291 (6 mg/kg) was given from day 5 until day 22.

In the LMC model with PC-9/ffluc cells, the mice in the control group became moribund within 28 days after tumor cell inoculation. Daily erlotinib treatment (25 mg/kg) remarkably delayed the progression of LMC, indicating that this dose of erlotinib was effective against LMC, which is consistent with its reported clinical activity [15]. Importantly, daily oral treatment with 6 mg/kg of AZD9291 further delayed the progression of LMC (Figure 3B, 3C). In parallel experiments, HCC827/luc and H1975/luc cells developed LMC, and AZD9291 showed efficacy against the LMC produced by HCC827/luc and H1975/luc cells (Figure 4). These observations clearly indicate that in addition to erlotinib, AZD9291 has activity against LMC of *EGFR*-mutant lung cancer.

### Effect of high dose of AZD9291 on LMC after acquiring EGFR-TKI resistance

**Figure 5:**
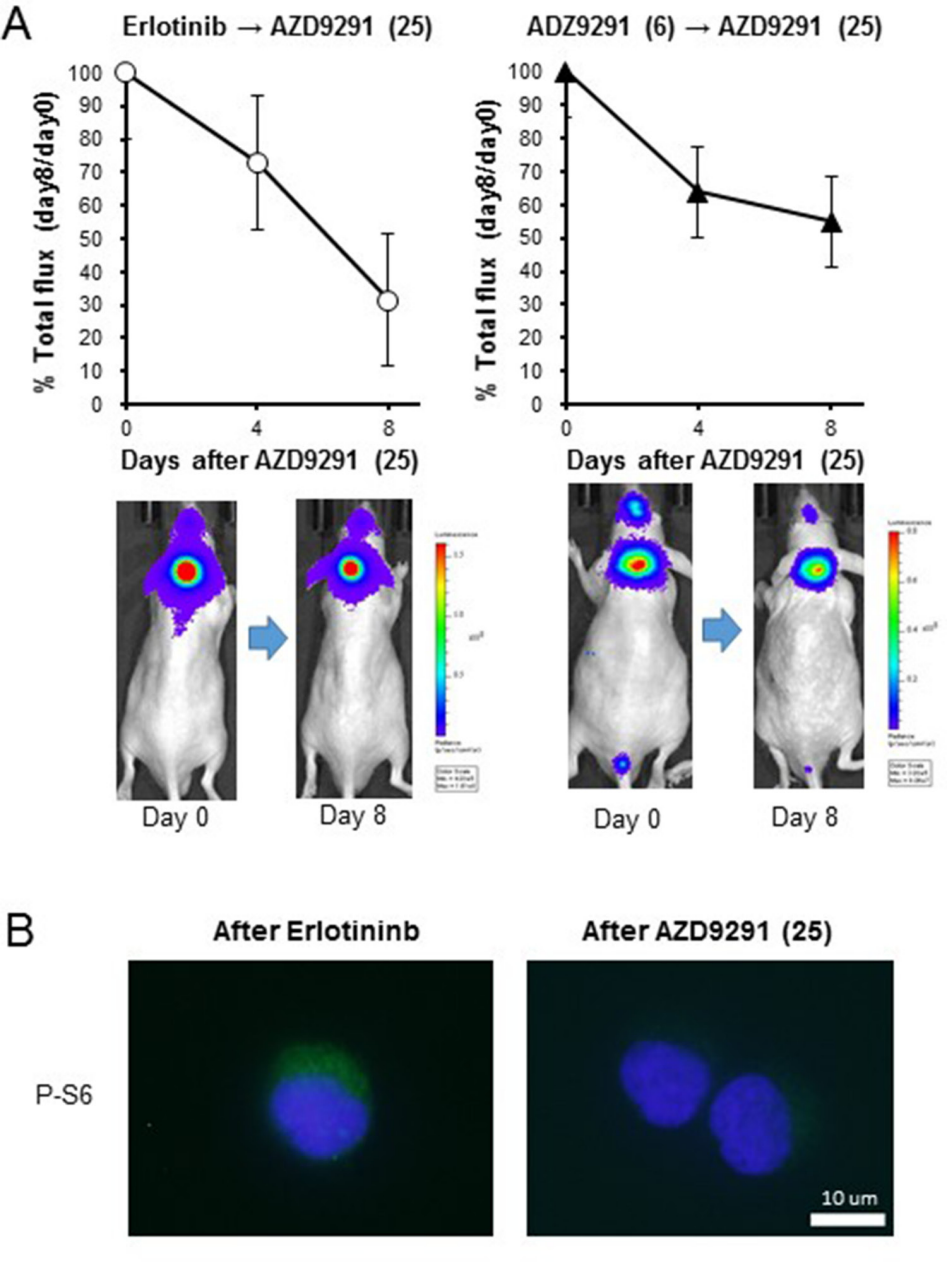
Effect of high dose-AZD9291 treatment after acquisition of EGFR-TKI resistance (**A**) After acquisition of resistance to treatment with 25 mg/kg of erlotinib (*N* = 5) or 6 mg/kg of AZD9291 (*N* = 5), the mice were given daily oral treatment with AZD9291 (25 mg/kg) for 8 days. The representative images are shown. (**B**) Cancer cells from the leptomeningeal space were collected after development of erlotinib resistance and high dose-AZD9291 treatment. The cell pellets were subsequently analyzed for S6 phosphorylation by immunofluorescence.

**Figure 6:**
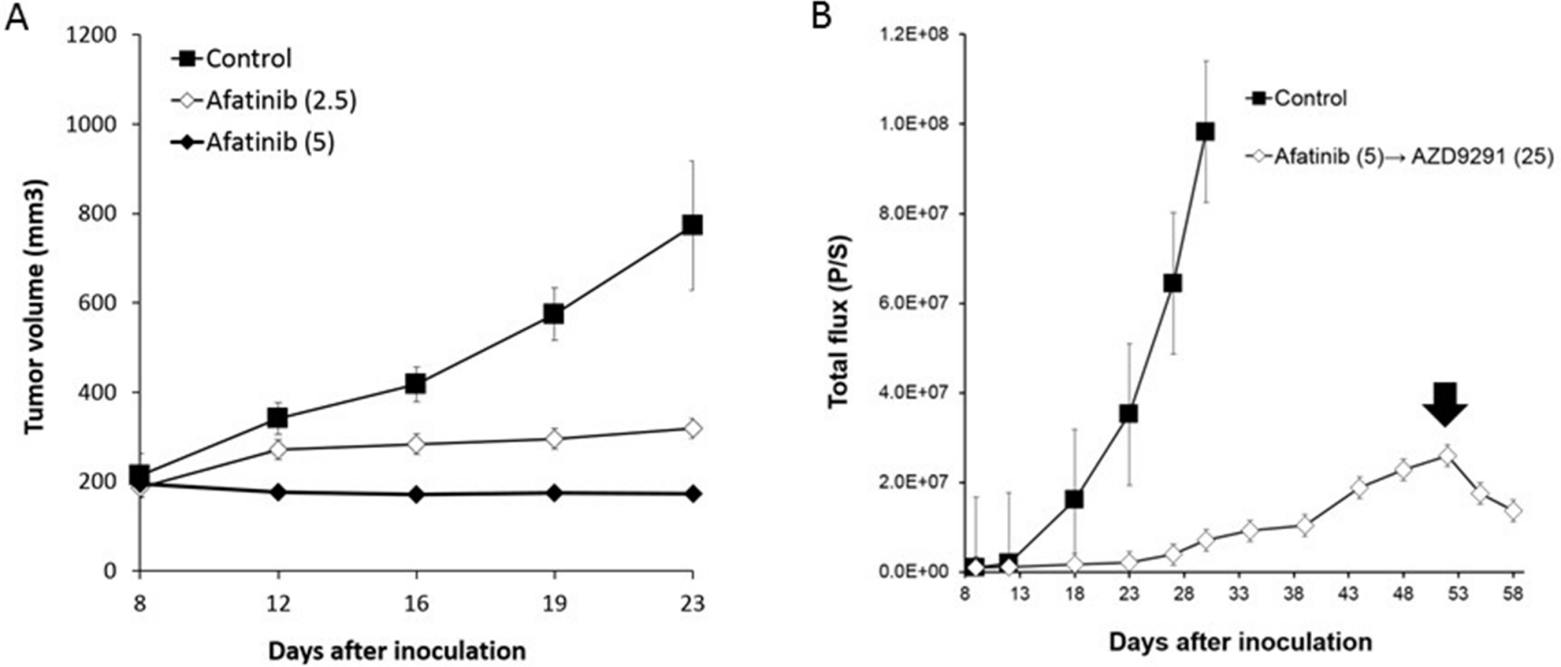
The efficacy of afatinib and high dose-AZD9291 after afatinib resistance in LMC model (**A**) PC-9/ffluc cells were inoculated subcutaneously in SHO-SCID mice. Mice were treated with 2.5 mg/kg (*N* = 5) or 5 mg/kg of afatinib (*N* = 5). Treatment was given daily for 23 days. Tumor volume was measured using calipers on the indicated days. Mean ± SE tumor volumes are shown. (**B**) LMC mice with PC-9/ffluc cells were administered afatinib (5 mg/kg) daily from day 8 until day 50. Luminescence was evaluated as total flux (p/s: photons/second). After resistance was acquired to afatinib, the LMC mice were administered AZD9291 (25 mg/kg) once daily for 8 days (from day 51 until day 58). Arrow indicates the initiation of the high dose AZD9291 (25 mg/kg) treatment.

We next sought to examine whether a higher dose of AZD9291 showed activity against an erlotinib-resistant LMC model. When the mice with progressed LMC by PC-9/ffluc cells after continuous treatment with 25 mg/kg of erlotinib were treated with a high dose of AZD9291 (25 mg/kg), the LMC was regressed (Figure 5A). The high dose-AZD9291 treatment also regressed LMC that had progressed after treatment with 6 mg/kg of AZD9291 (Figure 5A). Interestingly, phosphorylated S6 in leptomeningeal cancer cells was suppressed after a high dose of AZD9291, as determined by immunofluorescence analysis (Figure 5B). This confirmed the efficacy of high dose-AZD9291 treatment against LMC. In parallel experiments, we obtained similar results with LMC models treated with afatinib. Briefly, 5 mg/kg of afatinib, which could prevent the enlargement of subcutaneous PC-9/ffluc tumors (Figure 6A), slowed the progression of LMC. However, after continuous treatment with afatinib, LMC in the treated mice progressed. It should be noted that the high dose of ADZ9291 (25 mg/kg) could regress LMC refractory to afatinib treatment (Figure 6B).

### Toxicity profiles of EGFR-TKIs *in vivo*

**Figure 7:**
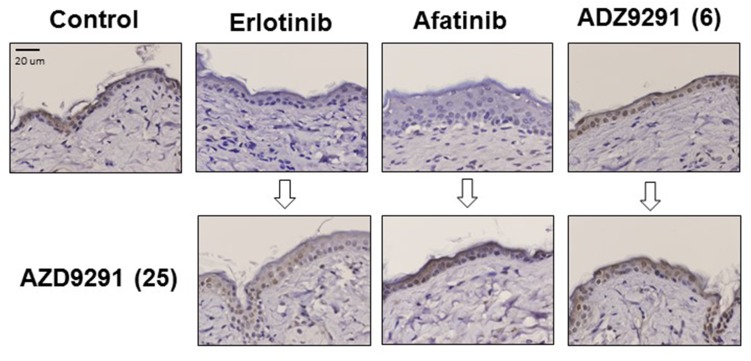
The effect of continuous EGFR-TKI treatment on MAPK phosphorylation in normal skin cells PC-9/ffluc cells were inoculated subcutaneously in SHO-SCID mice. Mice were treated daily with 25 mg/kg of erlotinib, 5 mg/kg of afatinib, or 6 mg/kg of AZD9291 for 50 days. Then, small skin tissues (3 mm × 3 mm) were harvested from the mice (upper panels). The mice were further treated with 25 mg/kg of AZD9291 for 8 days, and then small skin tissues (3 mm × 3 mm) were harvested again from them (lower panels). Phosphorylated ERK expression in these skin lesions was evaluated by immunohistochemistry. (x400).

To evaluate the toxicity of EGFR-TKI treatment, we measured the body weights of the mice during treatment. Treatment with EGFR-TKIs including administration of a high dose of AZD9291 did not caused severe body weight loss (Supplementary Figures 2, 3, 4). Furthermore, we assessed skin damage because AZD9291 has been reported to have less activity against wild type EGFR compared with mutant EGFRs such as exon 19 deletion, and L858R and T790M [15]. Accordingly, expression of phosphorylated mitogen-activated protein kinase (pMAPK), a downstream molecule of EGFR in normal skin, remained present after continuous treatment with 6 mg/kg of AZD9291, while it was inhibited by the continuous treatment with 25 mg/kg of erlotinib or 5 mg/kg of afatinib (Figure 7). Moreover, inhibition of EGFR phosphorylation due to treatment with erlotinib or afatinib could be reversed after continuous treatment with 25 mg/kg of AZD9291. These results strongly suggest that AZD9291 at the dose without wild-type EGFR inhibition is tolerable and sufficient for controlling LMC refractory to first and second generation EGFR-TKIs.

## DISCUSSION

In the present study, we have established an *in vivo* imaging LMC model of *EGFR*-mutant lung cancer and demonstrated that third generation EGFR-TKI, AZD9291, can manage LMC in our model.

A recent study reported promising results with AZD9291 treatment (response rate of 51%, and progression free survival of 8.2 months) in patients who had experienced radiographic progression after administration of at least one EGFR-TKI [11]. However, since the effect of AZD9291 specifically on CNS lesions including LMCs was not reported in the study, the activity of AZD9291 against LMC was unknown. Here, we clearly showed that AZD9291 has potential against EGFR-TKI naïve LMC of *EGFR*-mutant lung cancer cells. In addition, AZD9291 may also have activity against LMC which is resistant to first and second generation EGFR-TKIs. Based on our preclinical evidence, we posit that clinical trials are required to evaluate the efficacy of AZD9291 treatment in *EGFR*-mutant lung cancer patients with CNS metastasis including LMC with or without resistance to other EGFR-TKIs.

The ineffectiveness of EGFR-TKIs on CNS metastasis is thought to be due to insufficient penetration of EGFR-TKIs [5]. This is corroborated by the evidence that shows erlotinib, which has better penetration in CNS lesions than gefitinib, more effectively treats CNS metastasis resistant to gefitinib treatment [14, 16]. However, gefitinib is generally effective against EGFR-TKI naïve CNS metastasis in *EGFR*-mutant lung cancer patients, even though only 1% of gefitinib penetrates into the CNS lesions [4, 5, 16, 17]. These observations suggest the involvement of other mechanisms that result in the development of marginal resistance to a low concentration of EGFR-TKIs in CNS lesions. Unfortunately, we could not recover tumor cells from the LMC model. Thus, the molecular mechanism by which PC-9/ffluc cells acquire resistance to EGFR-TKI treatment remains unknown.

For LMC resistant to the daily standard EGFR-TKI treatment, no effective therapy has been established. Intermittent high dose EGFR-TKI and EGFR-TKI pulse therapy was proposed previously [18]. However, recent retrospective studies reported that there was no significant difference between the effect of a daily standard dose of erlotinib and high dose erlotinib pulse therapy on the overall survival of patients [19, 20]. We also examined the effect of gefitinib-pulse treatment in our LMC model. However, gefitinib-pulse treatment was not superior to daily gefitinib treatment in the control (Supplementary Figure 5).

Afatinib is an irreversible EGFR-TKI that has been approved in many countries including US, EU, and Japan. The results of the compassionate-use program of afatinib indicate that this drug is equally effective in *EGFR*-mutant lung cancer patients with CNS metastasis compared with the patients without CNS metastasis [7]. We found that afatinib at a dose of 5 mg/kg, which was almost equivalent to 25 mg/kg of erlotinib against subcutaneous tumors with PC-9/ffluc cells, remarkably delayed the progression of LMC in our model (Figure 5). The clinical efficacy of afatinib on CNS metastasis could be replicated in the LMC model, confirming clinical relevance of this model. Therefore, our LMC model may be useful for testing experimental and novel therapies for LMC.

In conclusion, we have established an LMC model for *EGFR*-mutant lung cancer and demonstrated the potential of third generation EGFR-TKI AZD9291, on not only EGFR-TKI naïve LMC but also EGFR-TKI-refractory LMC. Our preclinical evidence suggest therapeutic potential of AZD9291 on *EGFR*-mutant lung cancer patients with CNS metastasis including LMC with or without resistance to other EGFR-TKIs.

## MATERIALS AND METHODS

### Cell cultures and reagents

The *EGFR*-mutant human lung adenocarcinoma cell line PC-9 with deletions in *EGFR* exon 19 (del E746_A750) and HCC827, with deletions in *EGFR* exon 19 were purchased from Immuno-Biological Laboratories Co. (Gunma, Japan) and the American Type Culture Collection (Manassas, VA), respectively. H1975 cells, with the L858R/T790M double mutations in *EGFR*, were kindly provided from Drs. Yoshitaka Sekido (Aichi Cancer Center Research Institute, Japan) and John D. Minna (University of Texas Southwestern Medical Center). H1975/luc (JCRB1486) and HCC827/luc (JCRB1516) cells, which are luciferase transfected by Dr. Takashi Murakami (Laboratory of Tumor Biology, Takasaki University of Health and Welfare, Takasaki, Gunma, Japan), were provided from JCRB cell Bank (Osaka, Japan). PC-9 ffLuc-cp156 transfectant (PC-9/ffluc) was established as previously described [13]. These cells were maintained in RPMI-1640 medium supplemented with 10% fetal bovine serum (FBS) and antibiotics. All cells were passaged for less than 3 months before renewing frozen, early-passage stocks. Cells were regularly screened for mycoplasma contamination using MycoAlert Mycoplasma Detection Kits (Lonza, Rockland, ME). The cell lines were authenticated at the laboratory of the National Institute of Biomedical Innovation (Osaka, Japan) by short tandem repeat analysis in May 2015. Gefitinib, erlotinib, afatinib, and AZD9291 were obtained from Selleck Chemicals (Houston, TX).

### Cell viability assay

Cell viability was measured by the MTT dye reduction method. Tumor cells were plated at a density of 2 × 10^3^/100 μl per well in RPMI 1640 plus 10% FBS in 96-well plates and incubated for 24 hours. EGFR-TKIs were then added to each well, and incubation was continued for another 72 hours. Cell growth was measured with MTT solution (2 mg/mL; Sigma, St. Louis, MO), as described [21].

### Western blot

Protein aliquots of 25 μg each were resolved by SDS polyacrylamide gel (Bio-Rad, Hercules, CA) electrophoresis and transferred to polyvinylidene difluoride membranes (Bio-Rad). After washing thrice, the membranes were incubated with Blocking One (Nacalai Tesque, Inc., Kyoto, Japan) for 1 hour at room temperature and then incubated overnight at 4°C with primary antibodies against β-Actin, phospho-S6 (240/244), S6, phospho-EGFR (Y1068;Cell Signaling Technology, Beverly, MA), and human EGFR (R & D Systems, Minneapolis, MN). Subsequently, the membranes were washed thrice again, and incubated for 1 hour at room temperature with species-specific horseradish peroxidase–conjugated secondary antibodies. Immunoreactive bands were visualized with SuperSignal West Dura Extended Duration Substrate, an enhanced chemiluminescent substrate (Pierce Biotechnology, Rockford, IL). Each experiment was performed at least three times, independently.

### Tumor cell inoculation in severe combined immunodeficiency (SHO-Prkdc^scid^Hr^hr^) mice

We used 6-week-old female SHO-Prkdc^scid^Hr^hr^ mice (SHO-SCID mice: Charles River, Yokohama, Japan) for the study. For the subcutaneous tumor model, cultured tumor cells (PC-9/ffluc; 3 × 10^6^ cells/0.1 ml) were implanted subcutaneously into the flanks of each mouse. For the leptomeningeal metastasis model, the scalp was sterilized with 70% ethanol, and the cultured tumor cells were injected into the leptomeningeal space (between the external occipital protuberance and first cervical vertebra) with a 27G needle.

The size of subcutaneous tumors and body weights of the mice were measured twice per week, and tumor volume was calculated in mm^3^ (width^2^ × length/2). After 8 days, the mice were either left untreated or treated orally with gefitinib, erlotinib, afatinib, or AZD9291.

This study was carried out in strict accordance with the recommendations of the Guide for the Care and Use of Laboratory Animals by the Ministry of Education, Culture, Sports, Science, and Technology in Japan. The protocol was approved by the Committee on the Ethics of Experimental Animals and the Advanced Science Research Center, Kanazawa University, Kanazawa, Japan (approval no. AP-153499). All surgeries were performed on mice anesthetized with sodium pentobarbital, and efforts were made to minimize the suffering of the animals.

### Luciferase expression and radiographic analyses with the IVIS imaging system

After inoculation, the quantity of tumors was tracked in live mice by repeated noninvasive optical imaging of tumor-specific luciferase activity using the IVIS Lumina XR Imaging System (PerkinElmer, Alameda, CA). Twenty minutes after an intraperitoneal injection of the luciferase substrate luciferin (150 mg/kg), the mice were photographed under bright-field illumination and images were overlaid with luminescence data gathered over the maximum exposure period (5–30 seconds) in anesthetized by 2% isofluorane. The intensity of the bioluminescence signal was analyzed using Living Image 4.0 (PerkinElmer) by serially quantifying peak photon flux at the selected region of interest (ROI) covering the tumor. This was corrected for total area of ROI and time lapse during which the bioluminescence signals were picked by the CCD camera and expressed as photo/s/cm^2^/sr.

### Immunofluorescence

Mice brains harvested from mice inoculated with PC-9/ffluc cells into the leptomeningeal space were washed with PBS, and tumor cells was collected in a tube. The tubes were centrifuged at 800 rpm for 15 minutes and cell pellets were subsequently analyzed for S6 phosphorylation. Immunofluorescence analysis was performed as described earlier [22]. Briefly, cells on cover slips were fixed in 1% paraformaldehyde for 15 minutes, followed by treatment with 0.2% Triton X-100 on ice for 20 minutes. The coverslips were then incubated with rabbit phosphor S6 (240/244) antibody (Cell Signaling) overnight at 4°C, followed by incubation for 1 hour with Alexa 488-conjugated goat anti-rabbit (Molecular Probes). Confocal microscopy analysis was performed using an Olympus fluorescence microscope (Olympus, Japan).

### Immunohistochemistry of skin tissue samples

Phospho-p44/42 MAPK (Erk1/2, Thr202/Tyr204, Rabbit monoclonal antibody) was purchased from Cell Signaling Technology. Peroxidase-based immunohistochemistry detection system [EnVision+ System-HRP, Rabbit (DAB+)], antibody diluent, and serum-free protein block were purchased from Dako. Formalin-fixed paraffin-embedded tissue skin sections were subjected to antigen retrieval and endogenous peroxidase blocking, and were incubated with primary antibody overnight at 4°C. Following overnight incubation, slides were rinsed and incubated with a peroxidase-labeled polymer. The tissue sections were then rinsed and stained with 3,3′-diaminobenzidine (DAB) substrate-chromogen and then counterstained with Hematoxylin Gill I (EMD Millipore) and bluing reagent (EMD Millipore).

## SUPPLEMENTARY MATERIALS FIGURES


