# Supplementary material for: High efficacy of third generation EGFR inhibitor AZD9291 in a leptomeningeal carcinomatosis model with EGFR-mutant lung cancer cells
Source: Oncotarget. 2015 Dec 24;7(4):3847–56. doi: 10.18632/oncotarget.6758 (PMC4826174; doi:10.18632/oncotarget.6758)
Supplement: Supplementary file 1 [file oncotarget-07-3847-s001.pdf]

High efficacy of third generation EGFR inhibitor AZD9291 in a leptomeningeal carcinomatosis model with *EGFR*-mutant lung cancer cells

Supplementary Materials

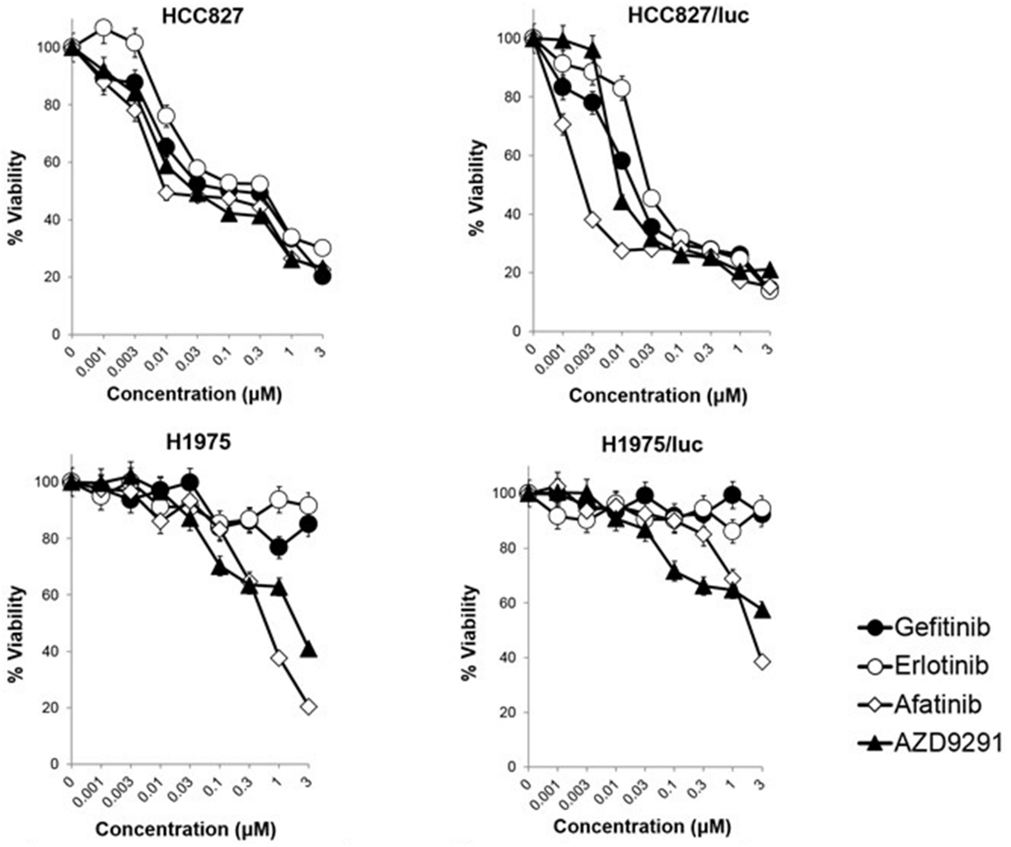

Supplementary Figure S1: Sensitivity of HCC827/luc and H1975/luc cells to EGFR-TKIs *in vitro*. HCC827, HCC827/luc, H1975, and H1975/luc cells (2×10^3 cells per well) were incubated with various concentrations of erlotinib, gefitinib, AZD9291, and afatinib for 72 hours. Cell growth was determined by the MTT assay. Bars represent SD.

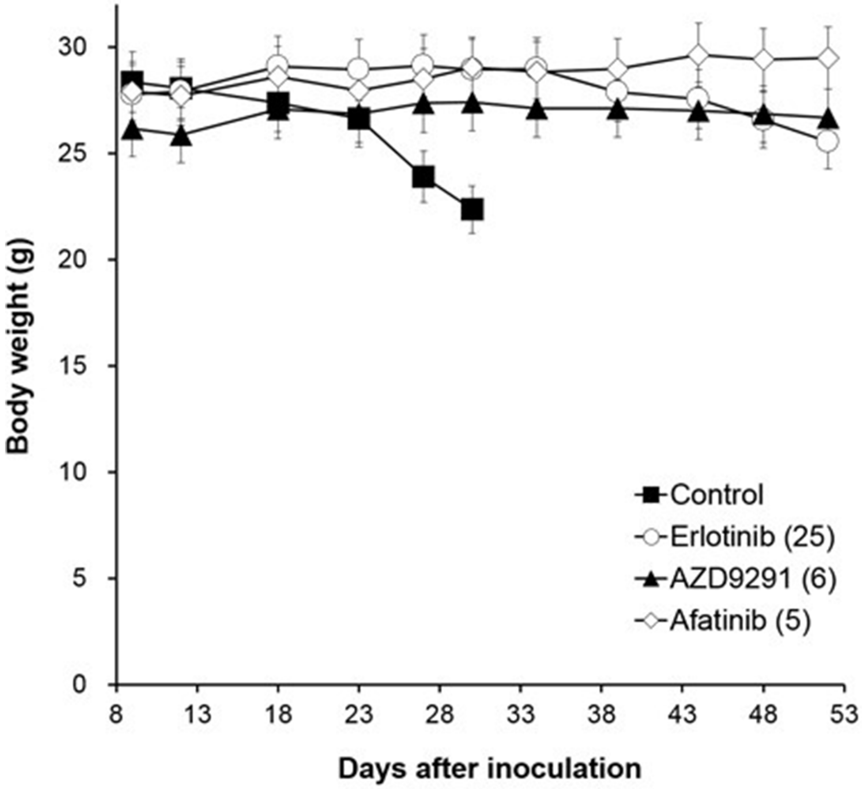

Supplementary Figure S2: Change in body weight of mice during EGFR-TKI treatment in the LMC model with PC-9/ ffluc cells. LMC mice with PC9/ffluc were administered erlotinib (25 mg/kg), afatinib (5 mg/kg), or AZD9291 (6 mg/kg) once daily for 44 days as in Figure 3B and Figure 5B, and their body weights were measured every 5 days.

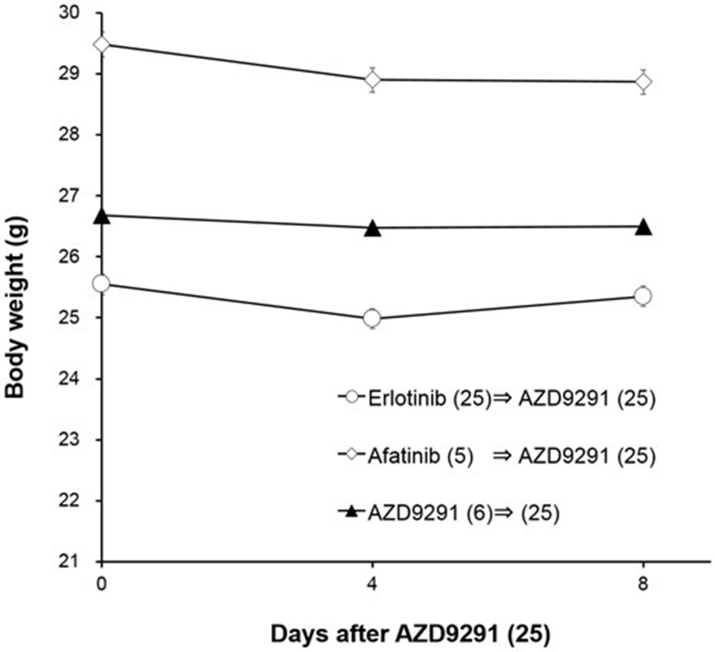

Supplementary Figure S3: Change in body weight of mice during 25 mg/kg AZD9291 treatment in the LMC model with PC-9/ffluc cells. The LMC mice with PC9/ffluc were switched to AZD9291 (25 mg/kg) treatment once daily for 8 days after acquiring resistance to erlotinib (25 mg/kg), afatinib (5mg/kg), or AZD9291 (6 mg/kg). Their body weights were measured every 4 days.

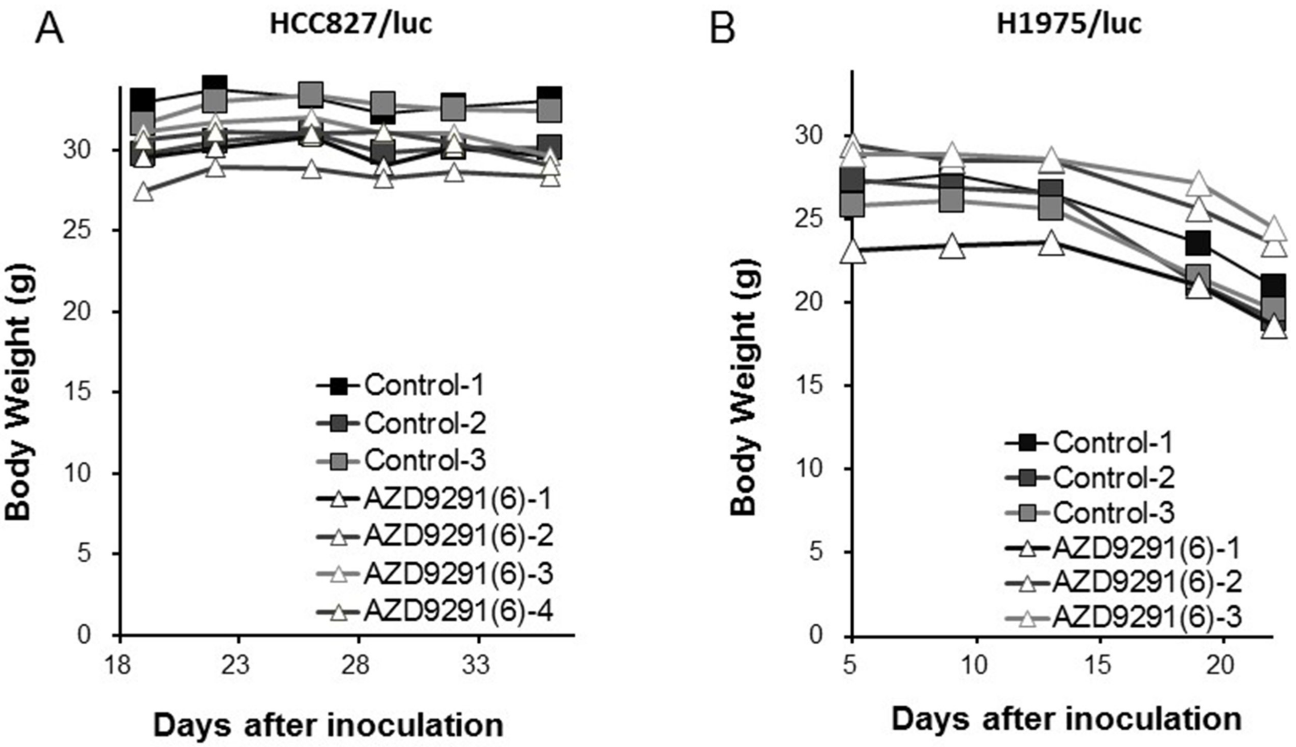

Supplementary Figure S4: Body weight of LMC mice during AZD9291 treatment. Body weight of LMC mice with HCC827/luc (**A**) and H1975/luc (**B**) were measured during AZD9291 (6 mg/kg) treatment.

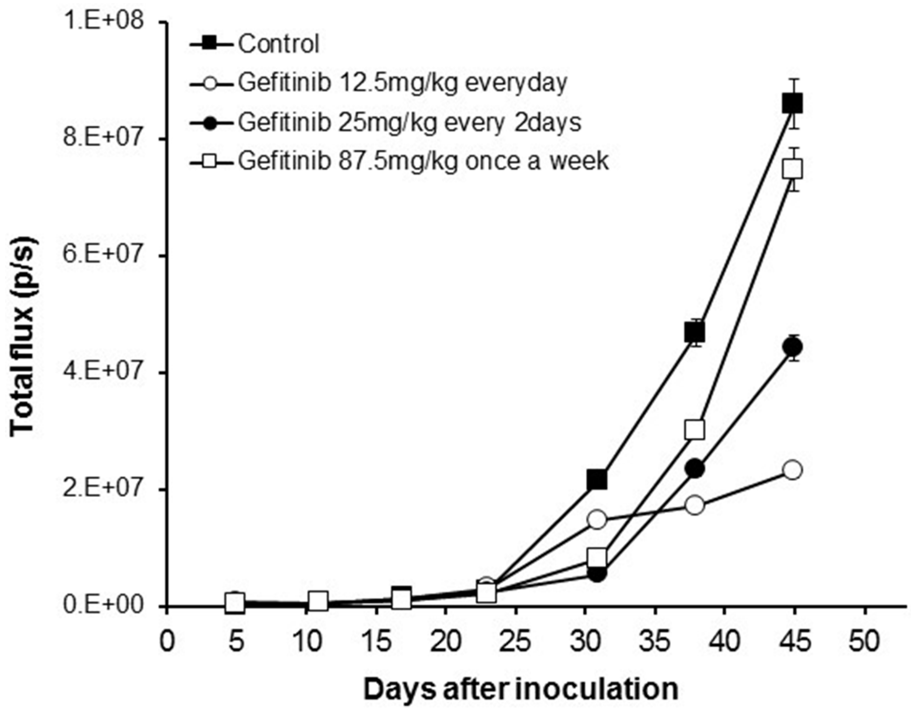

Supplementary Figure S5: Invalidity of pulsate high dose gefitinib therapies in LMC model with pC-9/ffluc cells. LMC mice with PC9/ffluc cells were administered gefitinib (12.5 mg/kg daily, 25 mg/kg every 2 days or 87.5 mg/kg once a week) for 6 weeks. Luminescence was evaluated as total flux (p/s: photons/second) and body weights were measured for groups of 4 mice every 5 days.
